# Global trends in antidepressant, atypical antipsychotic, and benzodiazepine use: A cross-sectional analysis of 64 countries

**DOI:** 10.1371/journal.pone.0284389

**Published:** 2023-04-26

**Authors:** Orges Alabaku, Alyssa Yang, Shenthuraan Tharmarajah, Katie Suda, Simone Vigod, Mina Tadrous

**Affiliations:** 1 University of Pittsburgh, Pittsburgh, PA, United States of America; 2 Leslie Dan Faculty of Pharmacy, University of Toronto, Toronto, ON, Canada; 3 Center for Health Equity Research and Promotion, VA Pittsburgh Healthcare System, Pittsburgh, PA, United States of America; 4 Department of Medicine, University of Pittsburgh, Pittsburgh, PA, United States of America; 5 Department of Medicine, University of Toronto, Toronto, ON, Canada; 6 Department of Psychiatry, Temerty Faculty of Medicine, University of Toronto, Toronto, ON, Canada; 7 Institute for Health Policy, Management and Evaluation, University of Toronto, Toronto, ON, Canada; 8 Women’s College Hospital and Women’s College Research Institute, Toronto, Ontario, Canada; 9 ICES, Toronto, ON, Canada; Ekiti State University College of Medicine, NIGERIA

## Abstract

**Objective:**

To describe the trends in use of antidepressants (ADs), atypical antipsychotics (AAPs), and benzodiazepines (BZDs) among high-, middle-, and low-income countries.

**Methods:**

A cross-sectional time-series analysis by country from July 2014 to December 2019 utilizing IQVIA’s Multinational Integrated Data Analysis database was conducted. Population-controlled rates of use were calculated in number of standard units of medications per drug class per population size. The United Nations’ 2020 World Economic Situation and Prospects was used to group countries into high-, middle-, and low-income. Percent change in rates of use per drug class was calculated from July 2014 to July 2019. Linear regression analyses were conducted to assess the predictability of percent change in use utilizing a country’s baseline rate of use per drug class and economic status as predictor variables.

**Results:**

A total of 64 countries were included: 33 high-, 6 middle-, and 25 low-income. Average baseline rates of use for ADs in high-, middle-, and low-income countries were 2.15, 0.35, and 0.38 standard units per population size, respectively. For AAPs, rates were 0.69, 0.15, and 0.13, respectively. For BZDs, rates were 1.66, 1.46, and 0.33, respectively. Average percent changes in use for ADs by economic status were 20%, 69%, and 42%, respectively. For AAPs, they were 27%, 78%, and 69%, respectively. For BZDs, they were -13%, 4%, and -5%, respectively. Some associations were found demonstrating that as a country’s economic status increases, percent change of AD (p = 0.916), AAP (p = 0.23), and BZD (p = 0.027) use decreases. Similarly, as baseline rate of use for ADs and AAPs increases, percent change in use decreases with p-values of 0.026 and 0.054, respectively. For BZDs, as baseline rate of use increases, percent change in use increases (p = 0.038).

**Conclusions:**

High-income countries have a higher rate of treatment utilization compared to low- and middle-income countries (LMICs) with treatment utilization increasing in all countries of interest.

## Introduction

The rising burden of mental illness continues to be a major global concern with steep costs and unmet needs for treatment worldwide. The World Health Organization (WHO) World Mental Health (WMH) surveyed across 28 countries estimated lifetime prevalence of DSM-IV anxiety, mood, disruptive behavior, and substance disorders for individuals at approximately 18–36% [1]. The 2020 Commonwealth Fund survey comparing mental health conditions and substance use between the U.S and 10 other high-income countries found significant differences in mental health diagnosis rates and treatment access [2]. The survey found that 23% of adults in the U.S. have received a mental health diagnosis, while adults in France, the Netherlands, and Germany had lower rates with 4%, 8%, and 9%, respectively. The survey also found that the U.S has a relatively low workforce capacity to meet mental health needs with only 105 professionals working in the mental health sector per 100,000 population. In the countries of interest, there are six high-income countries with more mental health professionals per 100,000 than the U.S. which include Norway, the Netherlands, France, Australia, Switzerland, and Canada. These findings highlight factors in variability of diagnosis and mental health capacity that may influence differences in mental health treatment utilization among high-income countries. When also considering low- and middle-income countries (LMICs), mental health treatment utilization may be even more variable between countries of different economic status due to inequities in treatment access which may be one factor that drives these differences.

LMICs comprise over 80% of the world’s population, and despite the high prevalence and impact of anxiety, mood fluctuations, impulse control, and substance use disorders identified in the WMH surveys within LMICs, more than 75% of individuals did not receive any care which demonstrates deficiencies in treatment access [3–10]. These deficiencies are further highlighted by the 2020 WHO Mental Health Atlas, a collection of global information about mental health resources, which found extreme variations between high- and low-income countries in the number of mental health facilities, mental health workers, and psychiatrists available [11]. The Mental Health Atlas reported 0.11 facilities, less than 2 mental health workers, and 0.1 psychiatrists per 100,000 population in low-income countries. These numbers were significantly lower compared to the 5.1 mental health facilities, over 60 mental health workers, and more than 8 psychiatrists per 100,000 population found in high-income countries. Given this extreme variability, there are ongoing efforts to improve treatment access and utilization within LMICs, including the World Health Assembly adopting the comprehensive Mental Health Action Plan 2013–2030 with four key objectives to improve mental health resources globally [12]. The objectives incorporate improving mental health leadership, promotion, prevention, and integrating mental health services in community settings. With these efforts to augment mental health treatment access and the current disparities that exist, it is important for studies to assess differences in treatment utilization and how continuing efforts such as these have impacted utilization patterns, especially among LMICs.

Antidepressants (ADs), atypical antipsychotics (AAPs), and benzodiazepines (BZDs) are among the most commonly utilized mental health treatments globally [13–19]. Since these medications became available on the market, rates of use among high-income countries have remained high over time [19–25]. Our study aims to expand on these findings and compare utilization trends between these medications across multiple countries of different economic status. As highlighted in the results and discussion, our study was able to achieve this aim.

## Materials and methods

### Data source

This study utilized data from IQVIA’s Multinational Integrated Data Analysis (MIDAS) database which consists of hospital and retail medication purchasing data for 66 countries between July 2014 to September 2020. Purchasing data was organized into standard units where one unit represents one package unit of a medication. The data did not contain information about individual facilities or patients who have purchased these drugs or obtained them from other sources.

### Study design

We conducted a repeated cross-sectional analysis to study global trends in AD, AAP, and BZD use between high- middle- and low-income countries from July 2014 to December 2019. There are three outcomes in this study: (1) differences in baseline rates of use per drug class; (2) differences in percent change in use per drug class from 2014 to 2019 in high-, middle-, and low-income countries; and (3) the predictive quality of percent change in use per drug class using a country’s economic status and baseline rates of use as predictors.

### Statistical analysis

From the total 66 countries available through the MIDAS database, 64 countries were included in the analysis. This is because countries that included Venezuela and Kuwait were missing a significant percentage of greater than 50% of their total purchasing data and were excluded. Purchasing data for each of the drug classes of interest were aggregated for the remaining countries. Missing purchasing data in a country for a specific AD, AAP, or BZD were excluded in the aggregated totals of that country. Purchasing data that occurred after December 2019 were excluded due to the start of the COVID-19 pandemic and the likely increase in mental health treatment utilization at that time. In addition, medications that did not fall into the drug classes of ADs, AAPs, or BZDs were also excluded along with herbal AD products. All included medications for each of the drug classes are provided in S4 Table.

The analysis group included 33 high-, 6 middle-, and 25 low-income countries. Countries were categorized as high-, middle-, and low-income based on the United Nations’ World Economic Situation and Prospects 2020 report’s classification criteria for developed, in-transition, and developing economies, respectively. Country population data was collected from the United Nations Department of Economic and Social Affairs. Data on average standard units purchased per drug class and per country between 2014 and 2019 were collected and included from IQVIA’s MIDAS database. Population-controlled baseline rates of use were calculated by dividing the average standard units per drug class between July 2014 to December 2019 by the country’s population between the same period. Population-controlled percent change in rates of use for the number of standard units purchased per drug class from July 2014 and July 2019 were also calculated. This was done by subtracting average population-controlled rates of use per drug class in 2014 from that of in 2019, dividing by the average population-controlled rate of use in 2014, and multiplying by 100. Lines of best fit and R-squared values were calculated per drug class using baseline rate of use on the x-axis and percent change in use on the y-axis. We conducted linear regression analyses to assess the predictability of percent change in use for ADs, AAPs, and BZDs. Linear regression analyses were conducted using Statistical Package for Social Sciences software (SPSS, IBM Corp.). Each country’s economic status and baseline rate of use were used as predictor variables to assess the predictability of percent change in use per drug class.

## Results

### Antidepressants

**Fig 1:**
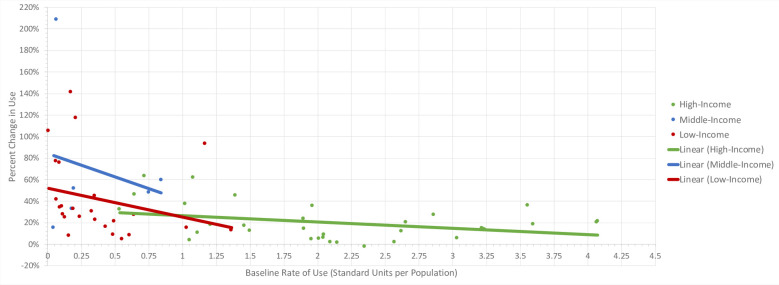
Antidepressant baseline rate of use vs. percent change in use in 64 countries, 2014–2019.

The average baseline rate of AD use across all 64 countries was 0.96 units per population. As seen in Fig 1, there is a general increase in the overall rate of AD use in the majority of countries included in the study cohort. On average, there is a percent change in use of 43% for ADs from 2014–2019. The rate of AD use is highest among high-income countries and lowest among LMICs. The average rates of use for ADs in high-, middle-, and low-income countries were 2.15, 0.35, and 0.38 standard units per population size, respectively. Average percent changes in the use for ADs in high-, middle-, and low-income countries were 20%, 69%, and 42%, respectively. When creating lines of best fit using baseline rate of use per drug class on the x-axis and percent change in use on the y-axis, R-squared values were 0.115 (p = 0.053), 0.048 (p = 0.676), and 0.067 (p = 0.212) for high-, middle-, low-income countries, respectively. In addition, linear regression analyses demonstrated an inverse relationship in that as a country’s economic status increases (p = 0.916) or baseline rate of use increases (p = 0.026), percent growth of AD use decreases (Table 1). As indicated by the p-value, a country’s economic status was found to be not significant in predicting percent change in use. However, as seen in Fig 1, LMICs tend to, on average, have lower baseline rates of AD use but higher percent growth in the use of ADs compared to high-income countries.

**Table 1:** Linear regression analyses of percent change in use for atypical antipsychotics, antidepressants, and benzodiazepines using a country economic status and baseline rate of use per drug class as predictors.

	Slope (Country economic status versus percent change in use)	P-Value	Slope (Baseline rate of use versus percent change in use)	P-Value
Antidepressants				
	-0.007	0.916	-0.122	0.026
Atypical Antipsychotics				
	-0.96	0.230	-0.421	0.054
Benzodiazepines				
	-0.063	0.027	0.045	0.038

Fig 1 demonstrates the relationship between antidepressant baseline rate of use and percent change in use in high-, middle-, and low-income countries of interest during the study period of 2014–2019.

### Atypical antipsychotics

**Fig 2:**
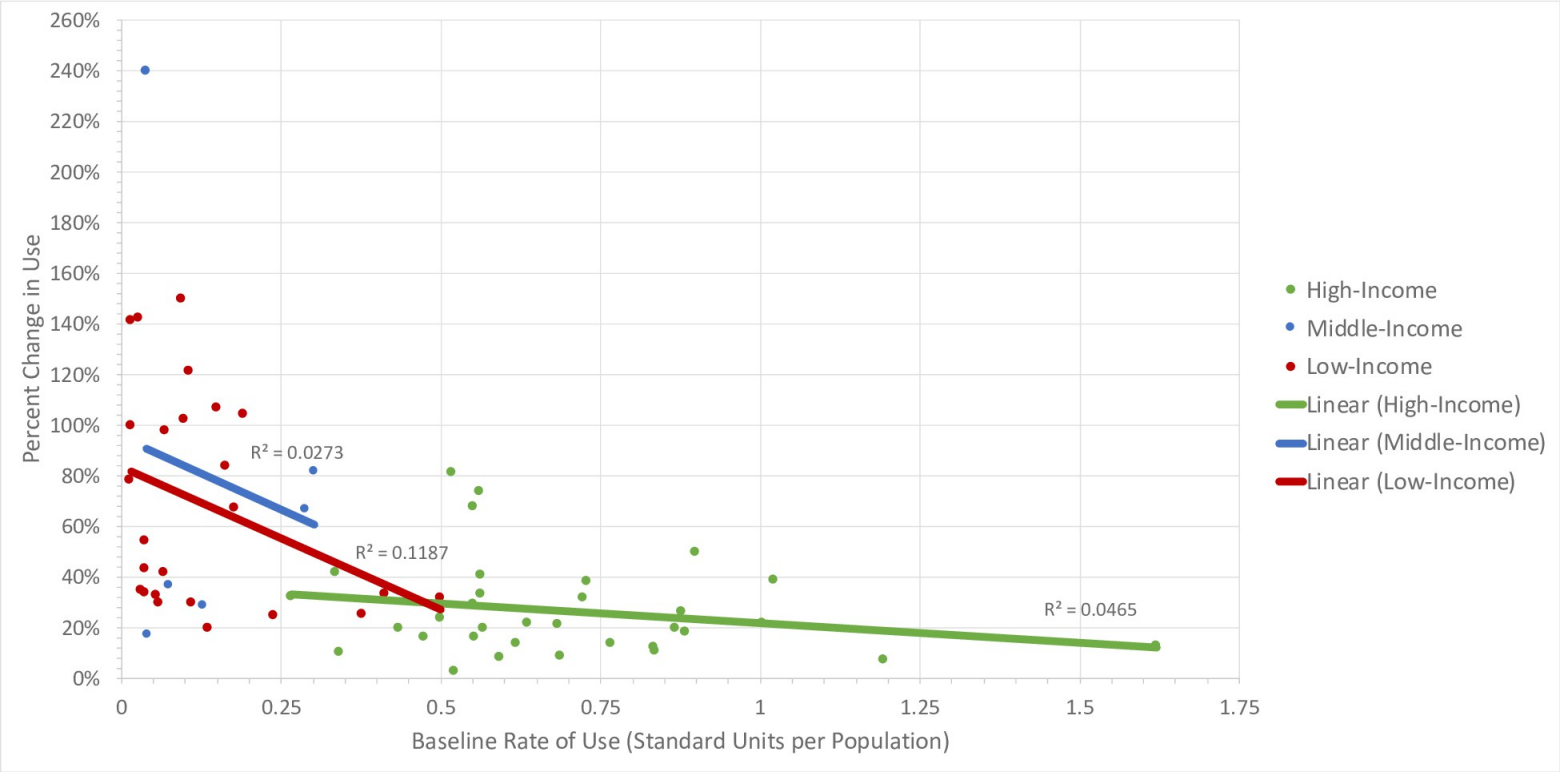
Atypical antipsychotic baseline rate of use vs. percent change in use in 64 countries, 2014–2019.

The average baseline rate of AAP use across all 64 countries was 0.32 standard units per population. Similar to ADs, Fig 2 demonstrates there is a general increase in the overall rate of AAP use in a majority of the countries included in the study cohort. The average percent change in AAP use was 58% between 2014–2019, with the highest and lowest use observed in high-income and LMICs, respectively. The average baseline rates of use were 0.69, 0.15, and 0.13 standard units per population size for high-, middle- and low-income countries, respectively. The average percent changes in use were 27%, 78%, and 69% for high-, middle-, and low-income countries, respectively. When creating lines of best fit, R-squared values were 0.047 (p = 0.228), 0.027 (p = 0.755), and 0.119 (p = 0.092) for high-, middle-, low-income countries, respectively. Linear regression analyses demonstrated the same inverse relationships as for ADs (Table 1). As a country’s economic status increases (p = 0.23) or baseline rate of use increases (p = 0.054), percent growth in AAP use decreases. As indicated by the p-values, a country’s economic status and baseline rate of AAP use were not significant predictors for precent change in AAP use. However, as seen in Fig 2, the trends are same to those of AD use. LMICs tend to, on average, have lower baseline rates of AAP use but higher percent growth in the use of AAPs compared to high-income countries.

Fig 2 demonstrates the relationship between atypical antipsychotic baseline rate of use and percent change in use in high-, middle-, and low-income countries of interest during the study period of 2014–2019.

### Benzodiazepines

**Fig 3:**
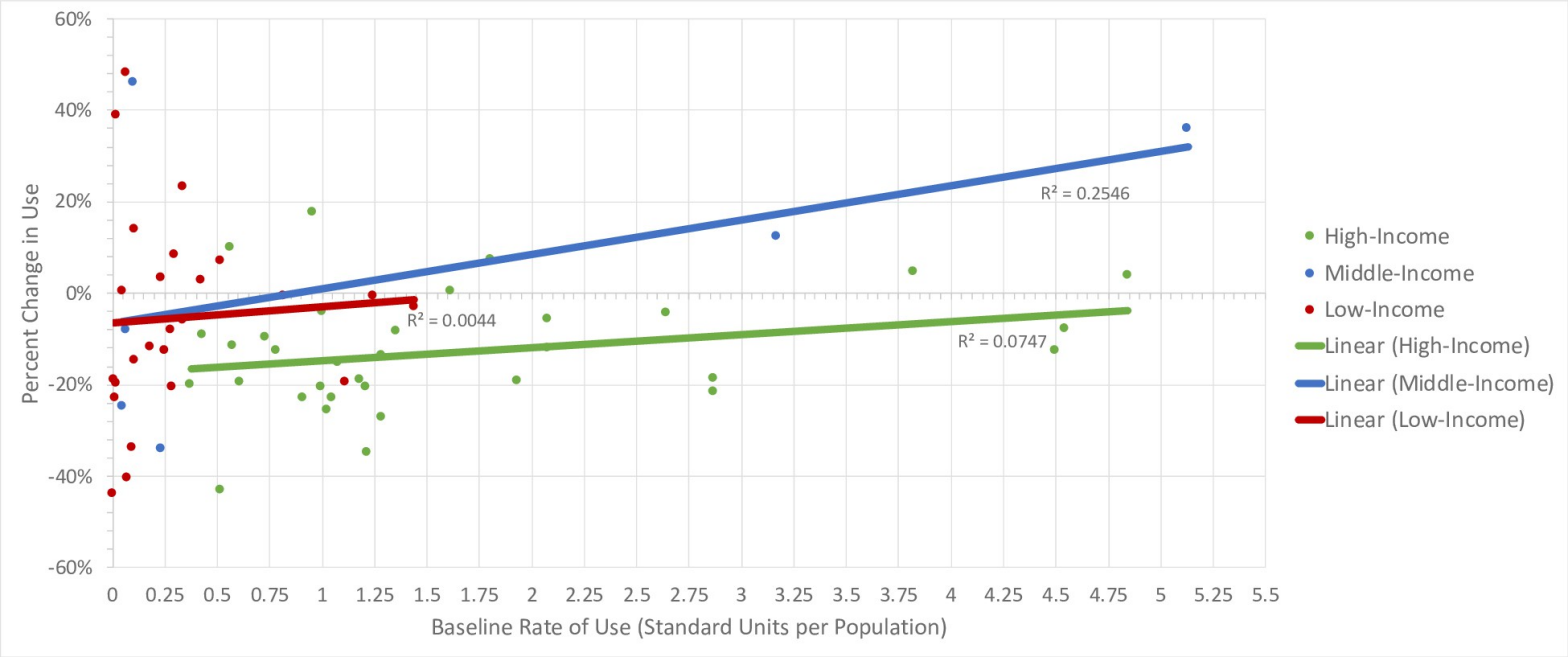
Benzodiazepine baseline rate of use vs. percent change in use in 64 countries, 2014–2019.

The average baseline rate of BZD use across all countries was 1.15 standard units per population size. In Fig 3, BZD use has increased in 16, decreased in 46, and remained the same in 2 countries, with an average percent change of -4.67% from 2014 to 2019. On average, BZD baseline rate of use was highest among high-income countries and lowest among LMICs. Average baseline rates of use for BZDs in high-, middle-, and low-income countries were 1.66, 1.46, and 0.33 standard units per population size, respectively. Average percent changes in use in high-, middle-, and low-income countries were -13%, 4%, and -5%, respectively. When creating lines of best fit, R-squared values were 0.075 (P = 0.124), 0.255 (P = 0.307), and 0.004 (P = 0.752) for high-, middle, low-income countries, respectively. As with ADs and AAPs, linear regression analyses demonstrated an inverse relationship between a country’s economic status (p = 0.027) and its percent change in use for BZDs (Table 1). However, contrary to the other drug classes, the linear regression analyses demonstrated a positive relationship between a country’s baseline rate of use and the percent change in use for BZDs (p = 0.038) (Table 1). Based on these p-values, both a country’s economic status and baseline rate of use were shown to be significant predictors for percent change in BZD use. These relationships indicate that high-income countries tend to have declining BZD rates of use. In addition, countries that have high baseline rates of BZD use have a small percent change and will continue to have high rates of use.

Fig 3 demonstrates the relationship between benzodiazepine baseline rate of use and percent change in use in high-, middle-, and low-income countries of interest during the study period.

## Discussion

It was determined that rates of use for all three drug classes were highest among high-income countries and lowest among LMICs. However, LMICs demonstrated higher rates of growth indicated by larger percent changes in the use of ADs and AAPs. To highlight these relationships, S1–S3 Tables summarize the specific baseline rates of use, percent changes in use, and absolute change in use for ADs, AAPs, and BZDs in each of the countries included. The inverse relationship when comparing economic status to baseline rate of use and percent change in use is especially apparent when comparing, for example, low-income countries such Mexico and Thailand to high-income countries such as the U.S. and Belgium. For ADs, Mexico and Thailand were found to have a baseline rate of use of 0.174 and 0.351 with a percent change in use of 141% and 45%, respectively. In contrast, the U.S. and Belgium were found to have a baseline rate of use of 3.597 and 3.034 with a percent change in use of 18% and 6%, respectively. These differences in utilization also similarly apply to AAPs with Mexico and Thailand having a baseline rate of use of 0.031 and 0.165 with a percent change in use of 35% and 84%, respectively. In contrast, the U.S. and Belgium have a much higher baseline rate of use of 3.597 and 0.619 with a lower percent change in use of 18% and 14%. Regarding benzodiazepines, results differ from ADs and AAPs which, on average, demonstrated increasing rates of use globally. Benzodiazepine rate of use, however, has decreased, especially for high-income countries which, historically, have had the highest rate of use. As shown in S3 Table, Mexico and Thailand were shown to have benzodiazepine baseline rates of use of 0.105 and 0.338 with a percent change in use of 14% and -6%. In contrast, the U.S. and Belgium have a higher baseline rate of use of 1.218 and 2.867 with a large decline in use with a percent change in use of -35% and -22%, respectively.

These results between high- and low-income countries suggest differences in mental health treatment utilization between countries of different economic status and between drug classes. Importantly, the results suggest that LMICs, with relatively low utilization rates of mental health treatments, are increasing use of ADs and AAPs to meet mental health needs. This increase in mental health treatment utilization is a potential reflection of the ongoing efforts to improve mental health treatment access globally. As reported in the 2020 WHO Mental Health Atlas, there have been improvements in increasing the number of countries with standalone policies for mental health, mental health promotion and prevention programs, and the number of mental health professionals from 2014–2020 [11]. However, the Mental Health Atlas reports continued significant differences between high- and low-income countries in the number of mental health facilities, mental health workers, and psychiatrists. In addition, socioeconomic factors also play a role in mental health disparities such as mental health stigma and low mental health literacy, which are major barriers to seeking mental health treatment [26, 27]. When considering that these factors are most prevalent among low-income countries, it further highlights the disparities in mental health treatment access and utilization among LMICs that goes beyond access to mental health facilities and workers.

When reviewing previous publications on the utilization of ADs and AAPs, a major limitation has been the small number of LMICs included in their analyses. To improve treatment access in the most vulnerable countries, the incorporation of LMICs is essential. Previously, Lewer et al. found that country-level healthcare spending as well as sociocultural norms and attitudes towards mental health were significant in predicting AD use among 27 European countries [28]. In addition, the Organization for Economic Co-Operation and Development (OECD) found that AD use was on the rise in all 29 developed countries of interest between the years of 2000 and 2017 [22]. The study also uncovered significant differences in AD use between countries with Iceland reporting AD consumption 10 times higher than that of Latvia. Similarly, Hálfdánarson et al. found that antipsychotic use increased in 10 of the 16 countries of interest from 2005–2014. This increase was represented as a percent change that ranged from 2.6% in the publicly insured population of the US to 91.2% in Colombia [19]. As previously mentioned on the underrepresentation of LMICs and as seen in similar studies, Taiwan was the only middle-income country included in their analysis while the rest of the countries included were high-income. Our study builds on these past findings derived primarily from high-income countries by also factoring in data from LMICs. This revealed that in addition to high-income countries, LMICs are also increasing their utilization of ADs and AAPs. Importantly, our study highlighted stark differences in drug utilization patterns between high-income countries and LMICs, as shown by large differences in rates of AD and AAP use as well as large differences in percent change in use for both drug classes. Our study highlighted that although LMICs, on average, have lower rates of AD and AAP use, they are experiencing, on average, higher rates of growth for each of the drug classes than high-income countries. These findings, specifically in LMICs, are a potential reflection of the ongoing efforts by organizations like the WHO to increase mental health treatment access in LMICs. However, given that significant differences in mental health treatment access still exist, efforts need to continue to improve treatment access among LMICs.

For BZDs, our study found that high- and low- income countries have experienced decreasing rates of use. This is expected because although BZDs have been proven effective in treating numerous conditions such as anxiety, agitation, and insomnia, their side effect profile is problematic [29]. One of the most significant side effects is the potential for misuse due to its addictive properties. In addition, BZD use in older adults has been shown to negatively affect cognitive functioning, increase the risk of falls, and increase mortality. Due to these side effects, medical guidelines recommend against the prescription of BZDs as first-line therapy in older adults [30]. However, studies conducted in the U.S., Switzerland, and other European countries found that BZDs are frequently prescribed to older adults [23, 31, 32]. In France, BZD prescription is especially high with more than 30% of older adults utilizing the dug [33]. This is reflective in our study, where France had one of the highest BZD rates of use globally. This country-level analysis of AAP, AD, and BZD use can further be refined to account for individual facility and patient characteristics. In addition, insights into differences in drug pricing and drug reimbursement policies between countries should be further explored to elucidate how these drug classes are used and accessed by populations. It is important to note that differences in pricing and reimbursement policies can vary by country, and that these variations need to be considered as they are a determinant in access to treatment. For example, in many high-income countries such as the U.K., Australia, Germany, and France, polices are in place that allow for the control of drug prices through government subsidies and drug buying agencies that negotiate drug prices [34, 35]. However, in the U.S, an exception to many high-income countries, and in some low-income countries, policies differ in that they allow for less control on drug pricing which results in an increase in the cost of treatment and a decrease in treatment access.

It is also important to mention that although the utilization of ADs and AAPs is increasing while the utilization of benzodiazepines is decreasing globally, this alone not does necessarily represent improvements in mental health care, but improvements in access. For example, as shown in this study, BZDs have relatively high utilization rates in high-income countries such as the U.S., France, and other European countries. However, these countries also have high rates benzodiazepine misuse such as the high utilization rates in older adults [23, 31–33]. This highlights that high utilization rates are not entirely reflective of rational drug use. Similarly, in LMICs where mental health treatment access is increasing, it is estimated by the WHO that four out of five people do not receive mental health or neurological care, and for those few who receive care, the treatment they receive is not entirely evidence based [36]. This is possibly a reflection of the short supply of trained mental health workers available in many LMICs to make effective and rational treatment decisions.

This study focuses on quantifying baseline trends in mental health treatment utilization prior to the COVID-19 pandemic. This is significant because the pandemic has resulted in increased anxiety and depression globally. As reported by the WHO, there was a 25% global increase in the prevalence of anxiety and depression within the first year of the COVID-19 pandemic [37]. The U.S. Census Bureau Household Pulse Survey comparing data from January to June 2019 and January 2021 found that during the pandemic, about 4 in 10 adults reported symptoms of anxiety and depression–an increase from 1 in 10 adults surveyed from January to June 2019 [38]. Similarly, analysis of data from the U.K. Household Longitudinal Study found increases in psychological distress throughout various time frames during the COVID-19 pandemic from 2019 to April 2020, there was an increase from 20.8% to 29.5%, while in January 2021, there was a similar increase to 27.1% [39]. Given the increase in mental health conditions, our study focuses only on pre-pandemic trends to avoid the likely increase in treatment utilization stemming from the pandemic. Therefore, the results of our study can be further built upon and refined to assess changes in treatment utilization trends pre-, during, and post-pandemic.

Our results are not without limitations. Firstly, IQVIA’s MIDAS dataset did not include information on individual facilities or patients, meaning that our analysis was not able to capture data beyond medication use at the country-level. Due to this limitation, this study could not take into account the demographic characteristics of the individuals using the medications of interests, and how differences in demographic characteristics between countries impacted the estimated utilization rates. Furthermore, due to the lack of patient characteristics, our study could not take into account indications for treatment. Given that ADs, AAPs, and BZDs can be used in the treatment of non-mental health conditions, it is possible that rates of ADs, AAPs, and BZDs use are not a reflection that these mental health medications are being used only in the setting of mental health. However, based on studies conducted in the U.S. and Canada that assessed prescription patterns by indication for each of the three drug classes have shown that, especially for ADs and AAPs, the most common indications for use are for mental health conditions [40–43]. It is also important to note that prescribing patterns by indication can differ between countries, and that country-specific analyses should be further explored. Secondly, while we grouped countries as high-, middle-, and low-income based on UN classification criteria for developed, in-transition, or developing economies, respectively, there may have been unmeasured differences between countries within each group. Lastly, there was an uneven distribution between the number of high-, middle-, and low-income countries included in this study, possibly skewing our data toward an overrepresentation of the 33 high-income and 25 low-income countries versus only 6 middle-income countries.

In this study, we determined baseline rates of use and percent changes in use of ADs, AAPs, and BZDs in high-, middle-, and low-income countries to analyze levels of treatment utilization per drug class in each country. We found that high-income countries demonstrated higher rates of treatment use for all three drug classes compared to LMICs, indicating that treatment may be marginalized in both low- and middle-income countries. In addition, we also found that the rate of treatment growth is highest in LMICs for ADs and BZDs, which, over time, may lower the disparities in treatment utilization between high- and LMIC. However, as discussed, due to potential differences between countries in demographic characteristics, rational use of mental health treatment, and the potential for ADs, AAPs, and BZDs to be used in non-metal health settings, our results cannot conclude that these trends are entirely reflective of rational mental health treatment utilization. In addition, these results may not reflect trends in other countries or for other mental health medications not included in this study. Moving forward, it is important for future research to explore these trends within countries, for a specific medication, and using other indicators for drug utilization such defined daily doses. Overall, our results suggest that there is a continuous need for analyzing global treatment utilization patterns to assess disparities in mental health treatment access, especially in LMICs.

## Supporting information

S1 Checklist STROBE statement—checklist of items that should be included in reports of observational studies. (DOCX)

S1 Table Low-, middle- and high-income countries and their respective population-controlled baseline rate of use, percent change in use, and absolute change in use for antidepressants. (DOCX)

S2 Table Low-, middle- and high-income countries and their respective population-controlled baseline rate of use, percent change in use, and absolute change in use for atypical antipsychotics. (DOCX)

S3 Table Low-, middle- and high-income countries and their respective population-controlled baseline rate of use, percent change in use, and absolute change in use for benzodiazepines. (DOCX)

S4 Table List of included antidepressant, atypical antipsychotic, and benzodiazepine medications ^a^. ^a^ The medications are listed by their active drug that can be used as an antidepressant, atypical antipsychotic, or benzodiazepine, respectively.
(DOCX)
