# Supplementary material for: Global trends in antidepressant, atypical antipsychotic, and benzodiazepine use: A cross-sectional analysis of 64 countries
Source: PLoS One. 2023 Apr 26;18(4):e0284389. doi: 10.1371/journal.pone.0284389 (PMC10132527; doi:10.1371/journal.pone.0284389)
Supplement: S1 Checklist — (DOCX) [file pone.0284389.s001.docx]

STROBE Statement—checklist of items that should be included in reports of observational studies

|  | Item No. | Recommendation | Page  No. | Relevant text from manuscript |
| --- | --- | --- | --- | --- |
| **Title and abstract** | 1 | (*a*) Indicate the study’s design with a commonly used term in the title or the abstract | 1 | “Global trends in antidepressant, atypical antipsychotic, and benzodiazepine use: A cross-sectional analysis of 64 countries.” |
|  |  | (*b*) Provide in the abstract an informative and balanced summary of what was done and what was found | 2-3 | “A cross-sectional time-series analysis by country from July 2014 to December 2019 utilizing IQVIA’s Multinational Integrated Data Analysis database was conducted.”  “Linear regression analyses were conducted to assess the predictability of percent change in use utilizing a country’s baseline rate of use per drug class and economic status as predictor variables.”  “High-income countries have a higher rate of treatment utilization compared to low- and middle-income countries (LMICs) with treatment utilization increasing in all countries of interest.” |
| Introduction | | | |  |
| Background/rationale | 2 | Explain the scientific background and rationale for the investigation being reported | 4-6 | “The rising burden of mental illness continues to be a major global concern with steep costs and unmet needs for treatment worldwide”  “LMICs comprise over 80% of the world’s population, and despite the high prevalence and impact of anxiety, mood fluctuations, impulse control, and substance use disorders identified in the WMH surveys within LMICs, more than 75% of individuals did not receive any care which demonstrates deficiencies in treatment access [3-10].”  “…there are ongoing efforts to improve treatment access and utilization within LMICs, including the World Health Assembly adopting the comprehensive Mental Health Action Plan 2013-2030 with four key objectives to improve mental health resources globally [12].”  “With these efforts to augment mental health treatment access and the current disparities that exist, it is important for studies to assess differences in treatment utilization and how continuing efforts such as these have impacted utilization patterns, especially among LMICs.” |
| Objectives | 3 | State specific objectives, including any prespecified hypotheses | 6 | “Our study aims to expand on these findings and compare utilization trends between these medications across multiple countries of different economic status.” |
| Methods | | | |  |
| Study design | 4 | Present key elements of study design early in the paper | 6 | “We conducted a repeated cross-sectional analysis to study global trends in AD, AAP, and BZD use across 64 different countries from July 2014 to December 2019. There are three outcomes in this study: (1) differences in baseline rates of use per drug class; (2) differences in percent change in use per drug class from 2014 to 2019 in high-, middle-, and low-income countries; and (3) the predictive quality of percent change in use per drug class using a country’s economic status and baseline rates of use as predictors.” |
| Setting | 5 | Describe the setting, locations, and relevant dates, including periods of recruitment, exposure, follow-up, and data collection | 6 | “We conducted a repeated cross-sectional analysis to study global trends in AD, AAP, and BZD use across 64 different countries from July 2014 to December 2019.”  “Purchasing data that occurred after December 2019 were excluded due to the start of the COVID-19 pandemic and the likely increase in mental health treatment utilization at that time.” |
| Participants | 6 | (*a*) *Cohort study*—Give the eligibility criteria, and the sources and methods of selection of participants. Describe methods of follow-up  *Case-control study*—Give the eligibility criteria, and the sources and methods of case ascertainment and control selection. Give the rationale for the choice of cases and controls  *Cross-sectional study*—Give the eligibility criteria, and the sources and methods of selection of participants | 7 | “From the total 66 countries available through the MIDAS database, 64 countries were included in the analysis. This is because countries that included Venezuela and Kuwait were missing a significant percentage of greater than 50% of their total purchasing data and were excluded. Purchasing data for each of the drug classes of interest were aggregated for the remaining countries.”  “In addition, medications that did not fall into the drug classes of ADs, AAPs, or BZDs were also excluded along with herbal AD products. All included medications for each of the drug classes are provided in S4 Table.” |
|  |  | (*b*) *Cohort study*—For matched studies, give matching criteria and number of exposed and unexposed  *Case-control study*—For matched studies, give matching criteria and the number of controls per case | n/a | n/a |
| Variables | 7 | Clearly define all outcomes, exposures, predictors, potential confounders, and effect modifiers. Give diagnostic criteria, if applicable | 6 | “There are three outcomes in this study: (1) differences in baseline rates of use per drug class; (2) differences in percent change in use per drug class from 2014 to 2019 in high-, middle-, and low-income countries; and (3) the predictive quality of percent change in use per drug class using a country’s economic status and baseline rates of use as predictors.” |
| Data sources/ measurement | 8* | For each variable of interest, give sources of data and details of methods of assessment (measurement). Describe comparability of assessment methods if there is more than one group | 6-7 | “This study utilized data from IQVIA’s Multinational Integrated Data Analysis (MIDAS) database which consists of hospital and retail medication purchasing data from 66 countries. Purchasing data was organized into standard units where one unit represents one package unit of a medication.”  “Countries were categorized as high-, middle-, and low-income based on the United Nations’ World Economic Situation and Prospects 2020 report’s classification criteria for developed, in-transition, and developing economies, respectively. Country population data was collected from the United Nations Department of Economic and Social Affairs. Data on average standard units purchased per drug class and per country between 2014 and 2019 were collected and included from IQVIA’s MIDAS database.” |
| Bias | 9 | Describe any efforts to address potential sources of bias | n/a | n/a |
| Study size | 10 | Explain how the study size was arrived at | 7 | “From the total 66 countries available through the MIDAS database, 64 countries were included in the analysis. This is because countries that included Venezuela and Kuwait were missing a significant percentage of greater than 50% of their total purchasing data and were excluded.” |

Continued on next page

| Quantitative variables | 11 | Explain how quantitative variables were handled in the analyses. If applicable, describe which groupings were chosen and why | 7-8 | “Population-controlled baseline rates of use were calculated by dividing the average standard units per drug class between July 2014 to December 2019 by the country’s population between the same period. Population-controlled percent change in rates of use for the number of standard units purchased per drug class from July 2014 and July 2019 were also calculated. This was done by subtracting average population-controlled rates of use per drug class in 2014 from that of in 2019, dividing by the average population-controlled rate of use in 2014, and multiplying by 100.” |
| --- | --- | --- | --- | --- |
| Statistical methods | 12 | (*a*) Describe all statistical methods, including those used to control for confounding | 7-8 | “Lines of best fit and R-squared values were calculated per drug class using baseline rate of use on the x-axis and percent change in use on the y-axis. We conducted linear regression analyses to assess the predictability of percent change in use for ADs, AAPs, and BZDs. Linear regression analyses were conducted using Statistical Package for Social Sciences software (SPSS, IBM Corp.). Each country’s economic status and baseline rate of use were used as predictor variables to assess the predictability of percent change in use per drug class.” |
|  |  | (*b*) Describe any methods used to examine subgroups and interactions | n/a | n/a |
|  |  | (*c*) Explain how missing data were addressed | 7 | “Missing purchasing data in a country for a specific AD, AAP, or BZD were excluded in the aggregated totals of that country.” |
|  |  | (*d*) *Cohort study*—If applicable, explain how loss to follow-up was addressed  *Case-control study*—If applicable, explain how matching of cases and controls was addressed  *Cross-sectional study*—If applicable, describe analytical methods taking account of sampling strategy | n/a | n/a |
|  |  | (*e*) Describe any sensitivity analyses | n/a | n/a |
| Results | | | | |
| Participants | 13* | (a) Report numbers of individuals at each stage of study—eg numbers potentially eligible, examined for eligibility, confirmed eligible, included in the study, completing follow-up, and analysed | 6 | “From the total 66 countries available through the MIDAS database, 64 countries were included in the analysis. This is because countries that included Venezuela and Kuwait were missing a significant percentage of greater than 50% of their total purchasing data and were excluded.”  “The analysis group included 33 high-, 6 middle-, and 25 low-income countries.” |
|  |  | (b) Give reasons for non-participation at each stage | n/a | n/a |
|  |  | (c) Consider use of a flow diagram |  |  |
| Descriptive data | 14* | (a) Give characteristics of study participants (eg demographic, clinical, social) and information on exposures and potential confounders | n/a | n/a |
|  |  | (b) Indicate number of participants with missing data for each variable of interest | n/a | n/a |
|  |  | (c) *Cohort study*—Summarise follow-up time (eg, average and total amount) | n/a | n/a |
| Outcome data | 15* | *Cohort study*—Report numbers of outcome events or summary measures over time | n/a | n/a |
|  |  | *Case-control study—*Report numbers in each exposure category, or summary measures of exposure | n/a | n/a |
|  |  | *Cross-sectional study—*Report numbers of outcome events or summary measures | 8-12 | “The average baseline rate of AD use across all 64 countries was 0.96 units per population. As seen in Fig 1, there is a general increase in the overall rate of AD use in the majority of countries included in the study cohort. On average, there is a percent change in use of 43% for ADs from 2014-2019. The rate of AD use is highest among high-income countries and lowest among LMICs. The average rates of use for ADs in high-, middle-, and low-income countries were 2.15, 0.35, and 0.38 standard units per population size, respectively. Average percent changes in the use for ADs in high-, middle-, and low-income countries were 20%, 69%, and 42%, respectively.”  “The average baseline rate of AAP use across all 64 countries was 0.32 standard units per population. Similar to ADs, Fig 2 demonstrates there is a general increase in the overall rate of AAP use in a majority of the countries included in the study cohort. The average percent change in AAP use was 58% between 2014-2019, with the highest and lowest use observed in high-income and LMICs, respectively. The average baseline rates of use were 0.69, 0.15, and 0.13 standard units per population size for high-, middle- and low-income countries, respectively. The average percent changes in use were 27%, 78%, and 69% for high-, middle-, and low-income countries, respectively.”  “The average baseline rate of BZD use across all countries was 1.15 standard units per population size. In Fig 3, BZD use has increased in 16, decreased in 46, and remained the same in 2 countries, with an average percent change of -4.67% from 2014 to 2019. On average, BZD baseline rate of use was highest among high-income countries and lowest among LMICs. Average baseline rates of use for BZDs in high-, middle-, and low-income countries were 1.66, 1.46, and 0.33 standard units per population size, respectively. Average percent changes in use in high-, middle-, and low-income countries were -13%, 4%, and -5%, respectively.” |
| Main results | 16 | (*a*) Give unadjusted estimates and, if applicable, confounder-adjusted estimates and their precision (eg, 95% confidence interval). Make clear which confounders were adjusted for and why they were included | 8-12 | Antidepressants: “When creating lines of best fit using baseline rate of use per drug class on the x-axis and percent change in use on the y-axis, R-squared values were 0.115 (p=0.053), 0.048 (p=0.676), and 0.067 (p=0.212) for high-, middle-, low-income countries, respectively. In addition, linear regression analyses demonstrated an inverse relationship in that as a country’s economic status increases (p=0.916) or baseline rate of use increases (p=0.026), percent growth of AD use decreases (Table 1).”  Atypical Antipsychotics: “When creating lines of best fit, R-squared values were 0.047 (p=0.228), 0.027 (p=0.755), and 0.119 (p=0.092) for high-, middle-, low-income countries, respectively. Linear regression analyses demonstrated the same inverse relationships as for ADs (Table 1). As a country’s economic status increases (p=0.23) or baseline rate of use increases (p=0.054), percent growth in AAP use decreases.”  Benzodiazepines: “When creating lines of best fit, R-squared values were 0.075 (P=0.124), 0.255 (P=0.307), and 0.004 (P=0.752) for high-, middle, low-income countries, respectively. As with ADs and AAPs, linear regression analyses demonstrated an inverse relationship between a country’s economic status (p=0.027) and its percent change in use for BZDs (Table 1). However, contrary to the other drug classes, the linear regression analyses demonstrated a positive relationship between a country’s baseline rate of use and the percent change in use for BZDs (p=0.038) (Table 1).” |
|  |  | (*b*) Report category boundaries when continuous variables were categorized | n/a | n/a |
|  |  | © If relevant, consider translating estimates of relative risk into absolute risk for a meaningful time period | n/a | n/a |

Continued on next page

| Other analyses | 17 | Report other analyses done—eg analyses of subgroups and interactions, and sensitivity analyses | n/a | n/a |
| --- | --- | --- | --- | --- |
| Discussion | | | | |
| Key results | 18 | Summarise key results with reference to study objectives | 12-13 | “It was determined that rates of use for all three drug classes were highest among high-income countries and lowest among LMICs. However, LMICs demonstrated higher rates of growth indicated by larger percent changes in the use of ADs and AAPs.”  “These results between high- and low-income countries suggest differences in mental health treatment utilization between countries of different economic status and between drug classes. Importantly, the results suggest that LMICs, with relatively low utilization rates of mental health treatments, are increasing use of ADs and AAPs to meet mental health needs.” |
| Limitations | 19 | Discuss limitations of the study, taking into account sources of potential bias or imprecision. Discuss both direction and magnitude of any potential bias | 17-18 | “Our results are not without limitations. Firstly, IQVIA’s MIDAS dataset did not include information on individual facilities or patients, meaning that our analysis was not able to capture data beyond medication use at the country-level. Due to this limitation, this study could not take into account the demographic characteristics of the individuals using the medications of interests, and how differences in demographic characteristics between countries impacted the estimated utilization rates. Furthermore, due to the lack of patient characteristics, our study could not take into account indications for treatment. Given that ADs, AAPs, and BZDs can be used in the treatment of non-mental health conditions, it is possible that rates of ADs, AAPs, and BZDs use are not a reflection that these mental health medications are being used only in the setting of mental health. However, based on studies conducted in the U.S. and Canada that assessed prescription patterns by indication for each of the three drug classes have shown that, especially for ADs and AAPs, the most common indications for use are for mental health conditions [40-43]. It is also important to note that prescribing patterns by indication can differ between countries, and that county specific analyses should be further explored. Secondly, while we grouped countries as high-, middle-, and low-income based on UN classification criteria for developed, in-transition, or developing economies, respectively, there may have been unmeasured differences between countries within each group. Lastly, there was an uneven distribution between the number of high-, middle-, and low-income countries included in this study, possibly skewing our data toward an overrepresentation of the 33 high-income and 25 low-income countries versus only 6 middle-income countries.” |
| Interpretation | 20 | Give a cautious overall interpretation of results considering objectives, limitations, multiplicity of analyses, results from similar studies, and other relevant evidence | 18-19 | “In this study, we determined baseline rates of use and percent changes in use of ADs, AAPs, and BZDs in high-, middle-, and low-income countries to analyze levels of treatment utilization per drug class in each country. We found that high-income countries demonstrated higher rates of treatment use for all three drug classes compared to LMICs, indicating that treatment may be marginalized in both low- and middle-income countries. In addition, we also found that the rate of treatment growth is highest in LMICs for ADs and BZDs, which, over time, may lower the disparities in treatment utilization between high- and LMIC. However, as discussed, due to potential differences between countries in demographic characteristics, rational use of mental health treatment, and the potential for ADs, AAPs, and BZDs to be used in non-metal health settings, our results cannot conclude that these trends are entirely reflective of rational mental health treatment utilization. In addition, these results may not reflect trends in other countries or for other mental health medications not included in this study. Moving forward, it is important for future research to explore these trends within countries, for a specific medication, and using other indicators for drug utilization such defined daily doses. Overall, our results suggest that there is a continuous need for analyzing global treatment utilization patterns to assess disparities in mental health treatment access, especially in LMICs.” |
| Generalisability | 21 | Discuss the generalisability (external validity) of the study results | 18-19 | “In addition, these results may not reflect trends in other countries or for other mental health medications not included in this study. Moving forward, it is important for future research to explore these trends within countries, for a specific medication, and using other indicators for drug utilization such defined daily doses.” |
| Other information | |  | | |
| Funding | 22 | Give the source of funding and the role of the funders for the present study and, if applicable, for the original study on which the present article is based | n/a | n/a |

*Give information separately for cases and controls in case-control studies and, if applicable, for exposed and unexposed groups in cohort and cross-sectional studies.

**Note:** An Explanation and Elaboration article discusses each checklist item and gives methodological background and published examples of transparent reporting. The STROBE checklist is best used in conjunction with this article (freely available on the Web sites of PLoS Medicine at http://www.plosmedicine.org/, Annals of Internal Medicine at http://www.annals.org/, and Epidemiology at http://www.epidem.com/). Information on the STROBE Initiative is available at www.strobe-statement.org.
