# Supplementary material for: Global trends in antidepressant, atypical antipsychotic, and benzodiazepine use: A cross-sectional analysis of 64 countries
Source: PLoS One. 2023 Apr 26;18(4):e0284389. doi: 10.1371/journal.pone.0284389 (PMC10132527; doi:10.1371/journal.pone.0284389)
Supplement: S1 Table — (DOCX) [file pone.0284389.s002.docx]

**S1 Table. Low-, middle- and high-income countries and their respective population-controlled baseline rate of use, percent change in use, and absolute change in use for antidepressants.**

a. *Low-income countries*

| **Country** | **Baseline rate of use** | **Percent change in use** | **Absolute Change** |
| --- | --- | --- | --- |
| Algeria | 0.327 | 30% | 0.084 |
| Argentina | 0.552 | 5% | 0.025 |
| Brazil | 1.168 | 93% | 0.808 |
| Chile | 0.607 | 8% | 0.049 |
| China | 0.062 | 77% | 0.037 |
| Colombia | 0.129 | 25% | 0.029 |
| Ecuador | 0.107 | 35% | 0.032 |
| Egypt | 0.211 | 117% | 0.160 |
| India | 0.112 | 28% | 0.026 |
| Jordan | 0.091 | 34% | 0.025 |
| South Korea | 0.491 | 21% | 0.098 |
| Lebanon | 0.639 | 27% | 0.156 |
| Mexico | 0.174 | 141% | 0.143 |
| Morocco | 0.238 | 25% | 0.052 |
| Pakistan | 0.190 | 33% | 0.050 |
| Peru | 0.064 | 42% | 0.021 |
| Philippines | 0.008 | 105% | 0.006 |
| Saudi Arabia | 0.159 | 8% | 0.012 |
| South Africa | 0.431 | 16% | 0.065 |
| Taiwan | 0.484 | 9% | 0.040 |
| Thailand | 0.351 | 45% | 0.131 |
| Tunisia | 0.353 | 23% | 0.074 |
| Turkey | 1.362 | 13% | 0.162 |
| UAE | 0.087 | 76% | 0.046 |
| Uruguay | 1.031 | 15% | 0.153 |

b. *Middle-income countries*

| **Country** | **Baseline rate of use** | **Percent change in use** | **Absolute Change** |
| --- | --- | --- | --- |
| Belarus | 0.196 | 52% | 0.086 |
| Bosnia and Herzegovina | 0.752 | 48% | 0.292 |
| Kazakhstan | 0.045 | 15% | 0.006 |
| Russia | 0.180 | 33% | 0.052 |
| Serbia | 0.841 | 60% | 0.380 |
| Ukraine | 0.067 | 208% | 0.068 |

c. *High-income countries*

| **Country** | **Baseline rate of use** | **Percent change in use** | **Absolute Change** |
| --- | --- | --- | --- |
| Australia | 3.230 | 14% | 0.444 |
| Austria | 2.349 | -2% | -0.056 |
| Belgium | 3.034 | 6% | 0.168 |
| Bulgaria | 0.534 | 32% | 0.149 |
| Canada | 4.073 | 21% | 0.805 |
| Croatia | 1.206 | 18% | 0.203 |
| Czech Republic | 1.896 | 24% | 0.390 |
| Denmark | 2.568 | 2% | 0.043 |
| Estonia | 1.077 | 62% | 0.517 |
| Finland | 2.622 | 12% | 0.308 |
| France | 2.008 | 5% | 0.098 |
| Germany | 2.044 | 6% | 0.121 |
| Greece | 1.960 | 36% | 0.592 |
| Hungary | 1.053 | 4% | 0.038 |
| Ireland | 2.860 | 27% | 0.688 |
| Italy | 1.498 | 12% | 0.171 |
| Japan | 1.120 | 11% | 0.116 |
| Latvia | 0.642 | 46% | 0.245 |
| Lithuania | 1.021 | 37% | 0.332 |
| Luxembourg | 1.955 | 5% | 0.089 |
| Netherlands | 2.045 | 9% | 0.180 |
| New Zealand | 3.238 | 13% | 0.416 |
| Norway | 2.145 | 1% | 0.028 |
| Poland | 1.389 | 45% | 0.517 |
| Portugal | 3.554 | 36% | 1.122 |
| Romania | 0.718 | 63% | 0.361 |
| Slovakia | 1.458 | 17% | 0.233 |
| Slovenia | 1.899 | 14% | 0.255 |
| Spain | 2.653 | 21% | 0.501 |
| Sweden | 3.215 | 15% | 0.445 |
| Switzerland | 2.093 | 2% | 0.040 |
| UK | 4.067 | 21% | 0.758 |
| US | 3.597 | 18% | 0.602 |
