# Supplementary material for: Global trends in antidepressant, atypical antipsychotic, and benzodiazepine use: A cross-sectional analysis of 64 countries
Source: PLoS One. 2023 Apr 26;18(4):e0284389. doi: 10.1371/journal.pone.0284389 (PMC10132527; doi:10.1371/journal.pone.0284389)
Supplement: S2 Table — (DOCX) [file pone.0284389.s003.docx]

**S2 Table. Low-, middle- and high-income countries and their respective population-controlled baseline rate of use, percent change in use, and absolute change in use for atypical antipsychotics.**

a. *Low-income countries*

| **Country** | **Baseline rate of use** | **Percent change in use** | **Absolute Change** |
| --- | --- | --- | --- |
| Algeria | 0.178 | 67% | 0.091 |
| Argentina | 0.240 | 25% | 0.054 |
| Brazil | 0.191 | 104% | 0.144 |
| Chile | 0.111 | 29% | 0.030 |
| China | 0.137 | 20% | 0.025 |
| Colombia | 0.027 | 142% | 0.022 |
| Ecuador | 0.038 | 54% | 0.016 |
| Egypt | 0.106 | 121% | 0.083 |
| India | 0.059 | 30% | 0.015 |
| Jordan | 0.038 | 34% | 0.010 |
| South Korea | 0.098 | 102% | 0.073 |
| Lebanon | 0.150 | 107% | 0.106 |
| Mexico | 0.031 | 35% | 0.009 |
| Morocco | 0.069 | 97% | 0.047 |
| Pakistan | 0.037 | 43% | 0.013 |
| Peru | 0.016 | 100% | 0.010 |
| Philippines | 0.014 | 78% | 0.009 |
| Saudi Arabia | 0.056 | 32% | 0.014 |
| South Africa | 0.067 | 42% | 0.024 |
| Taiwan | 0.377 | 25% | 0.083 |
| Thailand | 0.165 | 84% | 0.098 |
| Tunisia | 0.095 | 150% | 0.090 |
| Turkey | 0.413 | 33% | 0.114 |
| UAE | 0.016 | 141% | 0.013 |
| Uruguay | 0.500 | 32% | 0.125 |

b. *Middle-income countries*

| **Country** | **Baseline rate of use** | **Percent change in use** | **Absolute Change** |
| --- | --- | --- | --- |
| Belarus | 0.128 | 29% | 0.034 |
| Bosnia and Herzegovina | 0.302 | 82% | 0.180 |
| Kazakhstan | 0.041 | 17% | 0.006 |
| Russia | 0.073 | 37% | 0.025 |
| Serbia | 0.289 | 67% | 0.139 |
| Ukraine | 0.038 | 240% | 0.041 |

c. *High-income countries*

| **Country** | **Baseline rate of use** | **Percent change in use** | **Absolute Change** |
| --- | --- | --- | --- |
| Australia | 0.688 | 9% | 0.059 |
| Austria | 0.835 | 12% | 0.099 |
| Belgium | 0.619 | 14% | 0.080 |
| Bulgaria | 0.336 | 42% | 0.115 |
| Canada | 1.194 | 7% | 0.081 |
| Croatia | 0.684 | 21% | 0.131 |
| Czech Republic | 0.552 | 29% | 0.138 |
| Denmark | 0.884 | 18% | 0.147 |
| Estonia | 0.562 | 73% | 0.293 |
| Finland | 1.621 | 13% | 0.203 |
| France | 0.436 | 20% | 0.078 |
| Germany | 0.638 | 22% | 0.126 |
| Greece | 1.022 | 39% | 0.330 |
| Hungary | 0.474 | 16% | 0.071 |
| Ireland | 0.878 | 26% | 0.205 |
| Italy | 0.564 | 33% | 0.158 |
| Japan | 0.837 | 11% | 0.086 |
| Latvia | 0.518 | 81% | 0.313 |
| Lithuania | 0.552 | 68% | 0.290 |
| Luxembourg | 0.342 | 10% | 0.034 |
| Netherlands | 0.567 | 20% | 0.105 |
| New Zealand | 0.867 | 19% | 0.160 |
| Norway | 0.731 | 38% | 0.244 |
| Poland | 0.564 | 40% | 0.188 |
| Portugal | 0.900 | 50% | 0.366 |
| Romania | 0.267 | 32% | 0.080 |
| Slovakia | 0.500 | 24% | 0.108 |
| Slovenia | 1.005 | 22% | 0.198 |
| Spain | 0.724 | 32% | 0.200 |
| Sweden | 0.521 | 3% | 0.014 |
| Switzerland | 0.768 | 14% | 0.100 |
| UK | 0.555 | 16% | 0.081 |
| US | 0.593 | 8% | 0.046 |
