# Supplementary material for: Global trends in antidepressant, atypical antipsychotic, and benzodiazepine use: A cross-sectional analysis of 64 countries
Source: PLoS One. 2023 Apr 26;18(4):e0284389. doi: 10.1371/journal.pone.0284389 (PMC10132527; doi:10.1371/journal.pone.0284389)
Supplement: S3 Table — (DOCX) [file pone.0284389.s004.docx]

**S3 Table. Table 1. Low-, middle- and high-income countries and their respective population-controlled baseline rate of use, percent change in use, and absolute change in use for benzodiazepines.**

a. *Low-income countries*

| **Country** | **Baseline rate of use** | **Percent change in use** | **Absolute Change** |
| --- | --- | --- | --- |
| Algeria | 0.234 | 3% | 0.007 |
| Argentina | 1.110 | -20% | -0.244 |
| Brazil | 0.516 | 7% | 0.035 |
| Chile | 0.285 | -21% | -0.069 |
| China | 0.069 | 48% | 0.026 |
| Colombia | 0.014 | -23% | -0.004 |
| Ecuador | 0.019 | -20% | -0.004 |
| Egypt | 0.071 | -40% | -0.037 |
| India | 0.107 | -15% | -0.017 |
| Jordan | 0.095 | -34% | -0.036 |
| South Korea | 0.818 | -1% | -0.006 |
| Lebanon | 0.426 | 3% | 0.011 |
| Mexico | 0.105 | 14% | 0.014 |
| Morocco | 0.183 | -12% | -0.024 |
| Pakistan | 0.338 | 23% | 0.067 |
| Peru | 0.250 | -13% | -0.031 |
| Philippines | 0.006 | -19% | -0.001 |
| Saudi Arabia | 0.004 | -44% | -0.002 |
| South Africa | 0.297 | 8% | 0.024 |
| Taiwan | 1.244 | -1% | -0.009 |
| Thailand | 0.338 | -6% | -0.022 |
| Tunisia | 0.278 | -8% | -0.025 |
| Turkey | 0.051 | 0% | 0.000 |
| UAE | 0.020 | 39% | 0.007 |
| Uruguay | 1.440 | -3% | -0.046 |

b. *Middle-income countries*

| **Country** | **Baseline rate of use** | **Percent change in use** | **Absolute Change** |
| --- | --- | --- | --- |
| Belarus | 0.068 | -8% | -0.006 |
| Bosnia and Herzegovina | 3.171 | 12% | 0.373 |
| Kazakhstan | 0.048 | -25% | -0.014 |
| Russia | 0.234 | -34% | -0.098 |
| Serbia | 5.128 | 36% | 1.587 |
| Ukraine | 0.099 | 46% | 0.035 |

c. *High-income countries*

| **Country** | **Baseline rate of use** | **Percent change in use** | **Absolute Change** |
| --- | --- | --- | --- |
| Australia | 1.000 | -21% | -0.240 |
| Austria | 1.076 | -15% | -0.183 |
| Belgium | 2.867 | -22% | -0.688 |
| Bulgaria | 0.561 | 10% | 0.049 |
| Canada | 1.023 | -26% | -0.309 |
| Croatia | 4.845 | 4% | 0.184 |
| Czech Republic | 0.783 | -13% | -0.104 |
| Denmark | 0.515 | -43% | -0.288 |
| Estonia | 0.729 | -10% | -0.072 |
| Finland | 1.285 | -27% | -0.420 |
| France | 2.644 | -4% | -0.118 |
| Germany | 0.374 | -20% | -0.086 |
| Greece | 1.809 | 7% | 0.124 |
| Hungary | 4.496 | -13% | -0.603 |
| Ireland | 1.286 | -14% | -0.193 |
| Italy | 2.077 | -6% | -0.124 |
| Japan | 2.866 | -19% | -0.597 |
| Latvia | 1.005 | -4% | -0.042 |
| Lithuania | 1.933 | -19% | -0.403 |
| Luxembourg | 2.079 | -12% | -0.272 |
| Netherlands | 1.354 | -8% | -0.120 |
| New Zealand | 0.431 | -9% | -0.042 |
| Norway | 1.047 | -23% | -0.277 |
| Poland | 0.573 | -12% | -0.071 |
| Portugal | 4.543 | -8% | -0.385 |
| Romania | 0.955 | 17% | 0.155 |
| Slovakia | 1.613 | 0% | 0.000 |
| Slovenia | 1.180 | -19% | -0.248 |
| Spain | 3.824 | 5% | 0.173 |
| Sweden | 0.912 | -23% | -0.240 |
| Switzerland | 1.210 | -21% | -0.288 |
| UK | 0.612 | -20% | -0.134 |
| US | 1.218 | -35% | -0.498 |
