# Supplementary material for: Global trends in antidepressant, atypical antipsychotic, and benzodiazepine use: A cross-sectional analysis of 64 countries
Source: PLoS One. 2023 Apr 26;18(4):e0284389. doi: 10.1371/journal.pone.0284389 (PMC10132527; doi:10.1371/journal.pone.0284389)
Supplement: S4 Table — a The medications are listed by their active drug that can be used as an antidepressant, atypical antipsychotic, or benzodiazepine, respectively. (DOCX) [file pone.0284389.s005.docx]

S4 Table. List of included antidepressant, atypical antipsychotic, and benzodiazepine medications ^a^

| **Antidepressants** | **Atypical Antipsychotics** | **Benzodiazepines** |
| --- | --- | --- |
| Agomelatine | Amisulpride | Alprazolam |
| Amineptine | Aripiprazole | Bentazepam |
| Amitriptyline | Asenapine | Bromazepam |
| Amitriptylinoxide | Blonanserin | Brotizolam |
| Amoxapine | Brexpiprazole | Cinazepam |
| Brexanolone | Cariprazine | Cinolazepam |
| Bupropion | Clozapine | Clonazepam |
| Buptriptyline | Iloperidone | Clotiazepam |
| Citalopram | Lumateperone | Cloxazolam |
| Clomipramine | Lurasidone | Delorazepam |
| Dibenzepin | Melperone | Diazepam |
| Desipramine | Olanzapine | Estazolam |
| Desvenlafaxine | Paliperidone | Etizolam |
| Duloxetine | Perospirone | Fludiazepam |
| Dosulepin | Pimavanserin | Flunitrazepam |
| Doxepin | Quetiapine | Flurazepam |
| Escitalopram | Risperidone | Flutazolam |
| Esketamine | Sertindole | Flutoprazepam |
| Fluoxetine | Tiapride | Gidazepam |
| Fluvoxamine | Ziprasidone | Halazepam |
| Imipramine | Zotepine | Haloxazolam |
| Imipraminoxide |  | Ketazolam |
| Isocarboxazid |  | Loprazolam |
| Levomilnacipran |  | Lorazepam |
| Lofepramine |  | Lormetazepam |
| Maprotiline |  | Medazepam |
| Melitracen |  | Mexazolam |
| Mianserin |  | Midazolam |
| Milncipran |  | Nimetazepam |
| Mirtazapine |  | Nitrazepam |
| Moclobemide |  | Nordazepam |
| Nefazodone |  | Oxazepam |
| Nitroxazepine |  | Oxazolam |
| Nortriptyline |  | Phenazepam |
| Opiramol |  | Pinazepam |
| Paroxetine |  | Prazepam |
| Phenelzine |  | Quazepam |
| Pipofezine |  | Temazepam |
| Pirlindole |  | Tofisopam |
| Propizepine |  | Triazolam |
| Protriptyline |  |  |
| Quinupramine |  |  |
| Reboxetine |  |  |
| Selegiline |  |  |
| Sertraline |  |  |
| Setiptiline |  |  |
| Tianeptine |  |  |
| Tranylcypromine |  |  |
| Trazodone |  |  |
| Trimipramine |  |  |
| Venlafaxine |  |  |
| Vilazodone |  |  |
| Vortioxetine |  |  |
|  |  |  |
|  |  |  |
|  |  |  |
|  |  |  |
|  |  |  |
|  |  |  |
|  |  |  |
|  |  |  |
|  |  |  |
|  |  |  |
|  |  |  |
|  |  |  |

^a^ The medications are listed by the active drug that can be used as an antidepressant, atypical antipsychotic, or benzodiazepine, respectively.
